# Comparison of the Incidence of Capsular Formation in Two-Stage, Implant-Based Breast Reconstruction Using an Insertion Funnel and Sizer

**DOI:** 10.1155/2021/3898585

**Published:** 2021-07-09

**Authors:** Jun Hyeok Kim, Jung Hyeou Kim, Ahwon Lee, Suk-Ho Moon, Young-Joon Jun, Deuk Young Oh

**Affiliations:** ^1^Department of Plastic and Reconstructive Surgery, College of Medicine, The Catholic University of Korea, Seoul, Republic of Korea; ^2^Department of Hospital Pathology, Seoul St. Mary's Hospital, College of Medicine, The Catholic University of Korea, Seoul, Republic of Korea

## Abstract

**Purpose:**

Capsular formation around breast implants can produce various complications, including erythema, tenderness, discomfort, and breast deformation. Moreover, the capsule is thought to be correlated with breast implant-associated anaplastic large cell lymphoma. The proposed technique of capsule reduction can prevent some of these complications. Thus, the authors suggest a no-touch technique in two-stage, implant-based breast reconstruction. *Patients and Methods*. This single-center retrospective study evaluated the medical records and digitalized pathological slides of patients who underwent two-stage, implant-based breast reconstruction between February 2018 and May 2019. The selected patients were divided into group A and group B. Group A underwent a no-touch technique that included the following two steps: (1) using a sizer as the frame to create the submuscular and acellular dermal matrix (ADM) pocket for expander insertion and (2) inserting the expander through the funnel. After the second stage of implant insertion, the capsule was harvested for biopsy of the ADM, chest wall, and muscle.

**Results:**

This study included 33 breasts (31 patients): 18 in group A and 15 in group B. The capsular thicknesses of the ADM, the chest wall, and the muscle of group A were significantly thinner than those in group B. Pearson's correlation coefficient indicated negative correlations between capsular thickness and age; underlying disease; lesion side; interval of two-stage implant insertion; size of the expander; and radiotherapy, chemotherapy, or hormone therapy.

**Conclusion:**

To reduce the incidence of capsular formation following breast reconstruction using prostheses, a no-touch technique that uses a funnel and sizer to avoid implant contact is both efficient and beneficial.

## 1. Introduction

Immediate breast reconstruction following mastectomy for breast cancer is not only a safe and effective treatment but is also psychologically beneficial to patients [1–5]. The most common method of breast reconstruction is two-stage implantation, in which a tissue expander is inserted at the time of surgery and subsequently replaced with the permanent implant at a later date [6]. However, collagenous capsules can form around the implant due to a foreign body reaction [7–9].

Although most capsules will stabilize eventually [7], some progress to capsular contracture, which is the most common complication of the collagenous capsule; its symptoms include erythema, tenderness, discomfort, and deformed breast [8, 10]. Moreover, formation of a breast capsule has been correlated with development of breast implant-associated anaplastic large-cell lymphoma (BIA-ALCL) [9, 11–14].

Histological studies have revealed that capsule formation is related to the immune system [8, 10, 15]. Previous attempts to decrease the incidence of capsular contracture have sought to avoid an immune response by applying nipple shields [16], acellular dermal matrices (ADMs) [17], leukotriene antagonists [18], and antibiotic irrigation [19]. As part of these efforts, the present study developed a “no-touch technique” that includes creation of an implant pocket using an implant sizer and implant insertion through a funnel.

## 2. Materials and Methods

This single-center retrospective study evaluated the medical records and digitalized pathological slides of patients who underwent two-stage implant-based breast reconstruction between February 2018 and May 2019. The patients were selected according to the following criteria:

(1) Inclusion criteria
  - Diagnosis of breast cancer
  - Underwent two-stage prosthesis breast reconstruction
  - Postoperative evaluation including pathological examination of the capsule during second-stage surgery
(2) Exclusion criteria
  - Delayed breast reconstruction
  - Underwent one-stage prosthesis or autologous breast reconstruction
  - History of procedures affecting the breast tissue (augmentation, fat injection, or foreign body injection)
  - No perioperative data (demographic data, volume of expander, adjuvant therapy, and digitalized pathological slides)

The selected patients were divided into two groups. Group A underwent a no-touch procedure to exchange the implant, while group B patients were treated using the conventional technique to insert the permanent implant. The no-touch technique included the following two steps:

- Using a sizer as the frame to create the submuscular and ADM pocket for expander insertion
- Inserting the expander through the funnel

## 3. Operative Technique

All patients underwent nipple-sparing mastectomy performed by an oncological surgeon, followed by two-stage, implant-based breast reconstruction that included immediate total submuscular dual-plane placement of a tissue expander using ADM and later exchange for a permanent implant performed by a plastic surgeon who was experienced in implant-based breast reconstruction. Mentor expanders and permanent implants (Mentor Corporation, Santa Barbara, CA, USA) were used in all patients.

**Figure 1:**
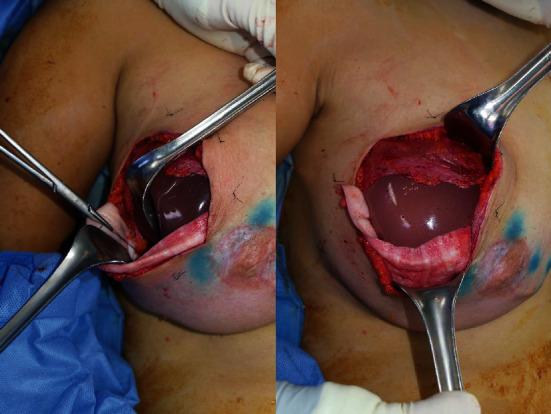
Intraoperative clinical photo: a disposable implant sizer was used to create the dual-implant breast pocket as the frame.

In group A, a no-touch technique was applied. After the oncological procedure, a submuscular pocket was formed by dissecting under the pectoralis muscle and placing a disposable sizer (Mentor Corporation) as the frame, and the ADM was sutured to create a dual-plane pocket for expander insertion. In this process, a curved pocket with a contour suitable for the expander and a permanent implant was created without contact of the expander with the environment or the hand of the surgeon prior to insertion (Figure 1).

After that, the pocket was rinsed with 2000 mg cefazolin, 80 mg gentamicin, and 500 mg Flagyl, and a tissue expander was placed beneath the dual-plane pocket composed of the pectoralis muscle and the ADM. When inserting the expander, a funnel (Art Funnel, Art Meditech, Seoul, Korea) was used to avoid exposure to the skin and the tunnel leading from the skin to the pocket. Finally, two drains were placed at the exit of the lateral margin of the inframammary fold: one in the pocket and the other subcutaneously.

These drains remained in place until output was <25 mL in 24 hours (within three weeks). All patients received a 1000 mg dosage of an antibiotic (a second-generation cephalosporin) 30 minutes before the incision was made and two additional doses in first 24 hours postoperatively. After surgery, all patients were instructed to wear a good-fitting sports bra.

### 3.1. Measurement of Capsular Thickness

**Figure 2:**
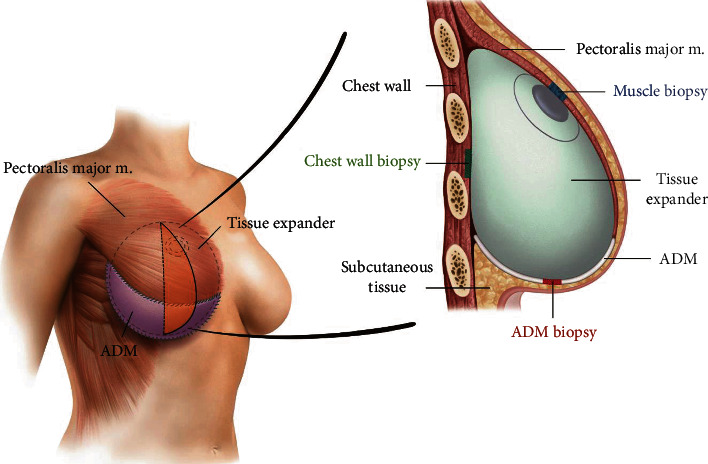
A schema of biopsy location: capsules that had formed on the ADM, chest wall, and muscle were harvested from the inside of the exposed pocket where the capsule was thickest as sections that measured 1 × 1 cm.

When replacing the expander with a permanent implant, the formed capsule was harvested, and the thickness was measured by the authors (including two clinicians and a pathologist) after preparation of histologic slides. An incision was made along the scars; the subcutaneous layer was dissected, and the expander was exposed by dividing the surface area where the muscle and ADM were fused. The expander was removed, and any capsules that had formed on the ADM, chest wall, or muscle were harvested from the inside of the exposed pocket where the capsule was thickest as sections that measured 1 × 1 cm (Figure 2).

**Figure 3:**
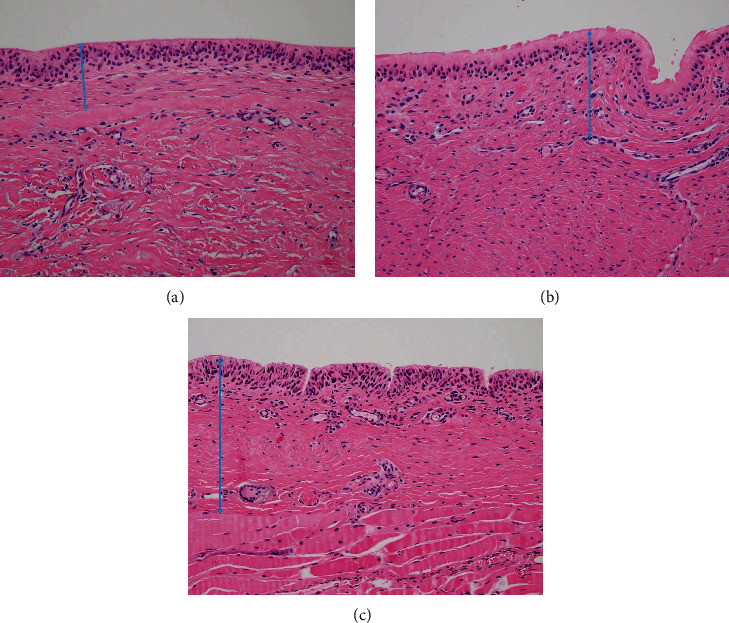
Pathology of capsular biopsies: (magnification ×100, capsular thicknesses indicated by double-headed arrow). (a) A capsule in the acellular dermal matrix layer. (b) A capsule at the chest wall. (c) A capsule in the muscle.

Capsular tissue was harvested in blocks, fixed in 10% buffered formalin, embedded in paraffin, cut into 5 *μ*m sections, and stained with hematoxylin and eosin. The stained sections were examined by a pathology scanner (Philips Ultra-Fast Scanner and Philips IntelliSite Pathology Solution, Philips Healthcare, USA) and digitalized. The digital slides were shared with the authors (including two clinicians and a pathologist), and each of whom performed measurements (Figure 3). Capsular thickness was determined at its thickest point, and the three measurements were averaged.

### 3.2. Statistical Analysis

For continuous variables, the mean and SD were used for description, and the difference between groups was compared using unpaired *t*-test for Gaussian distributions. One-way analysis of variance was used to compare the three columns, and Pearson's correlation analysis was performed to investigate the associations between capsular thickness and the variables of interest. A *p* value <0.05 indicated a statistically significant difference.

## 4. Results

A total of 33 breasts (31 patients) were included in this study. Group A was composed of 18 breasts, and group B comprised of 15 breasts. The baseline characteristics and demographic data of the patients are summarized in Table 1. The groups had no differences in age; underlying disease; lesion side; interval of two-stage implant insertion; size of the expander; or treatment with radiotherapy, chemotherapy, or hormone therapy. Capsular thicknesses of the ADM, the chest wall, and the muscle in group A were 0.114 ± 0.085 mm, 0.873 ± 0.263 mm, and 0.381 ± 0.142 mm, respectively. The same values in group B were 0.137 ± 0.060 mm, 1.153 ± 0.431 mm, and 0.493 ± 0.114 mm, respectively. All variables were statistically different between groups (*p* values < 0.001; Table 2). The capsular thicknesses of the ADM, the chest wall, and the muscle of group A were significantly thinner than those of group B (*p* value: 0.048, 0.029, and 0.020, respectively; Table 3). Pearson's correlation coefficient indicated negative correlations between capsular thickness and age; underlying disease; lesion side; interval of two-stage implant insertion; expander size; and use of radiotherapy, chemotherapy, or hormone therapy (Table 4).

## 5. Discussion

According to our results, the no-touch technique prevented peri-implant capsule thickening. The capsular thicknesses of the ADM, the chest wall, and the muscle of patients who underwent the no-touch technique were significantly thinner than those in patients treated with the conventional procedure (*p* value < 0.05). Moreover, age; underlying disease; lesion side; interval of two-stage implant insertion; size of the expander; and any concurrent radiotherapy, chemotherapy, or hormone therapy did not affect this outcome. That our no-touch technique produced significantly fewer peri-implant capsules supports the hypothesis that subclinical infection by coagulase-negative staphylococci (including *S. Epidermis*) and production of biofilm around implants led to formation of capsules and associated complications [20, 21]. A Baker Class IV capsular contracture in particular demonstrated a significantly greater incidence of tenderness with a distorted breast and implants with positive bacterial culture results [21].

Within each group, the capsular thicknesses of the ADM, the chest wall, and the muscle were statistically different (*p* values < 0.001). The ADM capsules were the thinnest, followed by capsules on the chest wall; muscle capsules were the thickest. It has been reported that capsule production can be avoided in ADM-covered implants [22, 23]. Considering that muscles typically demonstrate good blood flow and more frequent cell migration than in the chest wall, the likelihood of capsule thickening due to increase in extensive collagen deposition and increased myofibroblasts, neutrophils, macrophages, lymphocytes, and fibroblasts [8, 15, 23] is greater in muscle.

Immediate breast reconstruction using two-stage implantation is the most common approach, but peri-implant capsules can be created due to the foreign body reaction [7, 8]. Some capsules are expected to progress to capsular contracture that produces clinical complications of erythema, discomfort, tenderness, and a distorted breast [8]. And patients who develop capsular contracture with clinical signs (including a Baker Score of III or IV) demonstrate a significantly thicker capsule than patients with a Baker Score of I or II [8, 24]. Revision surgery is required to address a deformed breast caused by capsular contracture [15, 25]. In addition, formation of a breast capsule is considered to be associated with development of ALCL [9–12].

Histological studies have indicated that capsule formulation is related with the immune system, and macrophages, lymphocytes, and fibroblasts are predominant in the capsule [8, 15]. Published studies have verified methods to avoid immune responses to implantation, including application of nipple shields [16], ADM [17], leukotriene antagonist [18], and antibiotic washes [19].

A no-touch technique reduces the incidence of capsular creation by avoiding bacterial infection of the surgical field because it decreases the contact of expenders with the environment. First, the method of creating a dual-plane pocket using a sizer drastically reduces exposure of the expander to the subcutaneous tissue, muscle, and surgeon's hand. The conventional method involves creation of a suitable pocket for insertion or repeat insertion/removal of the expander in and out of the pocket. In contrast, the sizer eliminates potential bacterial contact due to its no-touch technique. After all manipulations required for pocket creation are complete, the area is rinsed thoroughly with triple antibiotic solution, and the expander is inserted after being cleaned. During expander insertion, use of a funnel prevents the expander from being exposed to the skin, the tunnel from the skin to the pocket, and the surgeon's hand. This no-touch technique reduces a series of exposures to the environment because the expander is fitted into the prepared pocket while being rinsed with antibiotic solution.

The average duration of implantation in the present study was 163.1 ± 82.4 days, which is relatively short, and capsular thickness is positively correlated with duration of implantation [8, 24]. However, considering the large impact of initial local reaction to implantation on formation of a capsule [8, 24], a no-touch technique to reduce capsular formation in the short-term will reduce symptomatic capsular contracture of a Baker Score of III or IV, even in the long term.

BIA-ALCL is a discrete type of T-cell lymphoma commonly implicated in capsule or peri-implant seroma [11], and the incidence has been estimated between 1 : 1000 and 1 : 30,000 women with textured implants used for both aesthetic and reconstructive purposes. In most cases, BIA-ALCL is confined to the implant capsule, and complete capsulectomy alone is the treatment of choice [14]. Kim et al. suggested that BIA-ALCL is associated with the peri-implant capsule on histologic examination either with or without capsular inflammation [13]. In addition, Evans et al. revealed that a persistent immune response to the bacteria on the capsule around the implant could produce an inflammatory microenvironment in which BIA-ALCL can occur [12]. Thus, we anticipate that our no-touch technique can decrease the incidence of BIA-ALCL.

## 6. Conclusion

In two-stage, implant-based breast reconstruction, a no-touch technique that includes use of a funnel and sizer to avoid touching the implant is efficient and beneficial at reducing capsular formation during breast reconstruction using prostheses. This technique can decrease the incidence of complications including capsular contracture with clinical symptoms or emergence of breast implant-associated ALCL.

## Figures and Tables

**Table 1:** Patient characteristics and demographic data.

	Group A	Group B	*p* value
Age (year)	44.94 ± 5.18	46.20 ± 9.88	0.792
Underlying diseases^†^	6 (37.5%)	3 (20%)	0.433
Lesion side			0.491
Right	8 (44.4%)	10 (55.6%)	
Left	9 (60.0%)	6 (40.0%)	
Interval (day)	140.6 ± 48.1	190.1 ± 108.4	0.426
Expander size (mL)	350.3 ± 94.7	358.3 ± 98.95	0.757
Radiotherapy	3 (16.7%)	4 (26.7%)	0.674
Chemotherapy	4 (25.0%)	6 (40.0%)	0.458
Hormone therapy	11 (68.8%)	11 (73.3%)	>0.999

^†^Underlying diseases include hypertension, hyperthyroidism, ovarian cyst, B-viral hepatitis, atopic dermatitis, gall bladder polyp, and Still's disease.

**Table 2:** Capsular thickness within each group.

	Capsular thickness (mm)			*p* value
	ADM	Chest wall	Muscle	
Group A	0.114 ± 0.085	0.873 ± 0.263	0.381 ± 0.142	<0.001^∗∗∗^
Group B	0.137 ± 0.060	1.153 ± 0.431	0.493 ± 0.114	<0.001^∗∗∗^
Total	0.125 ± 0.074	1.000 ± 0.372	0.432 ± 0.140	<0.001^∗∗∗^

Abbreviation: ADM: acellular dermal matrix. ^∗∗∗^*p* value is significant at <0.001.

**Table 3:** Capsular thickness: a comparison of groups A and B.

	Group A	Group B	*p* value
Capsular thickness (mm)			
ADM	0.114 ± 0.085	0.137 ± 0.060	0.048^∗^
Chest wall	0.873 ± 0.263	1.153 ± 0.431	0.029^∗^
Muscle	0.381 ± 0.142	0.493 ± 0.114	0.020^∗^

Abbreviation: ADM: acellular dermal matrix. ^∗^*p* value is significant at the 0.05 level.

**Table 4:** Correlation between the variables of interest and capsular thickness.

Variables	ADM		Chest wall		Muscle	
	Pearson correlation coefficient	*p* value	Pearson correlation coefficient	*p* value	Pearson correlation coefficient	*p* value
Age	-0.230	0.199	0.156	0.387	-0.008	0.966
Underlying Dz	0.294	0.097	-0.235	0.189	-0.100	0.580
Lesion side	0.031	0.864	0.012	0.945	-0.030	0.866
Interval	-0.298	0.092	0.022	0.902	0.218	0.222
Expander	0.104	0.565	-0.093	0.608	0.123	0.495
Radiotherapy	-0.165	0.360	0.030	0.869	0.231	0.197
Chemotherapy	-0.291	0.100	-0.094	0.604	0.080	0.660
Hormone Tx	-0.049	0.785	-0.070	0.700	-0.188	0.295

Abbreviation: ADM: acellular dermal matrix; Tx: therapy; Dz: diseases.

## Data Availability

Data are available on request.
